# Microcystins Alter Chemotactic Behavior in *Caenorhabditis elegans* by Selectively Targeting the AWA Sensory Neuron

**DOI:** 10.3390/toxins6061813

**Published:** 2014-06-10

**Authors:** Caroline E. Moore, Pamela J. Lein, Birgit Puschner

**Affiliations:** Department of Molecular Biosciences, School of Veterinary Medicine, 1089 Veterinary Medicine Drive, 2225 VM3B, University of California, Davis, Davis, CA 95616, USA; E-Mails: cemoore@ucdavis.edu (C.E.M.); pjlein@ucdavis.edu (P.J.L.)

**Keywords:** *Caenorhabidititis elegans*, chemotaxis, generalized linear model, microcystins, neurotoxicity, protein phosphatase, sensory neurons

## Abstract

Harmful algal blooms expose humans and animals to microcystins (MCs) through contaminated drinking water. While hepatotoxicity following acute exposure to MCs is well documented, neurotoxicity after sub-lethal exposure is poorly understood. We developed a novel statistical approach using a generalized linear model and the quasibinomial family to analyze neurotoxic effects in adult *Caenorhabditis elegans* exposed to MC-LR or MC-LF for 24 h. Selective effects of toxin exposure on AWA *versus* AWC sensory neuron function were determined using a chemotaxis assay. With a non-monotonic response MCs altered AWA but not AWC function, and MC-LF was more potent than MC-LR. To probe a potential role for protein phosphatases (PPs) in MC neurotoxicity, we evaluated the chemotactic response in worms exposed to the PP1 inhibitor tautomycin or the PP2A inhibitor okadaic acid for 24 h. Okadaic acid impaired both AWA and AWC function, while tautomycin had no effect on function of either neuronal cell type at the concentrations tested. These findings suggest that MCs alter the AWA neuron at concentrations that do not cause AWC toxicity via mechanisms other than PP inhibition.

## 1. Introduction

Microcystins (MCs) are toxins of global environmental concern, contaminating surface, ground, brackish, and marine waters [1,2]. Produced by cyanobacteria, there is increasing interest in these well-established acute hepatotoxins [3,4,5] as potential neurotoxins. In the aftermath of a tragic incident in 1996, when dialysis patients in Caruaru, Brazil, were inadvertently exposed to treatment water contaminated with MCs, 116 of the 131 sick patients (89%) reported general neurological symptoms including dizziness, vertigo, tinnitus, mild deafness, and, in severe cases, visual disturbances, blindness, and grand mal convulsions [6,7,8]. MCs are cyclic heptapeptides with two variable amino acids, and the over 80 different MC variants can exhibit differing physicochemical, toxicokinetic and toxicodynamic properties. MC-LR (which contains leucine and arginine in the variable amino acid positions) was the first MC to be chemically identified and is associated with most incidences of toxicity [9]. Therefore, MC-LR has been the focus of most diagnostic tests and experimental studies. The primary mechanism by which MCs cause acute hepatotoxicity is inhibition of serine/threonine protein phosphatases (PPs) 1 and 2A, [10,11] as a result of binding to the catalytic site of these holoenzymes.

Tight regulation of PP1 and PP2A is critical for normal neuron development and function [12,13], and dysregulation of PPs can alter synaptic plasticity and memory formation, contributing to neurological disorders such as Parkinson’s and Alzheimer’s diseases [14,15]. This suggests the possibility that MCs may cause neurotoxicity through interactions with PPs in neuronal cells. Cellular uptake of MCs occurs via organic anion transporter peptides (OATPs), which has been well documented in hepatocytes, and more recently demonstrated in the blood-brain-barrier, blood-cerebrospinal-fluid-barrier, and in human gliomas, glia cells and primary mouse neurons [16,17,18,19,20,21]. MC-LR and MC-RR cross the blood-brain-barrier in fish and cause behavioral defects [22,23], and intracerebroventricular administration of MC-LR causes cognitive dysfunction in rats [24], potentially via inhibition of hippocampal long-term potentiation [25]. Two hydrophobic MCs, MC-LF and MC-LW, are more potent than MC-LR at inhibiting PPs, and this correlates with their relative potency in causing neurodegeneration in primary neuron-glia co-cultures and primary mouse neurons [26,27]. Yet, whether MC exposure *in vivo* can cause neurotoxicity independent of neurodegeneration via targeted effects on specific neuronal cell types has yet to be determined. To develop a platform to address this question, we employed the *Caenorhabditis elegans* (*C. elegans*) as a model system.

*C. elegans* are a well-established neurotoxicological and neurological disease research model [28,29,30,31]. All 302 *C. elegans* neurons have been mapped and correlated to specific behaviors [32], including 32 presumed chemosensory neurons [33]. The AWA and AWC neurons are similar to vertebrate olfactory neurons in detecting volatile odors [34] and their signaling pathways have been used to study regulation of synaptic transmission and plasticity and memory [35,36] through the use of chemotaxis assays. Genetic ablation studies have shown the AWA and AWC sensory neurons are required for chemotaxis towards diacetyl and benzaldehyde, respectively, at the low concentrations used in this study [34,37]. In addition, pathway differences between olfactory adaptation (diminished chemosensory response after prolonged odor exposure) and transduction and neuron morphology are well established for the AWA and AWC sensory neurons, making it a suitable platform to investigate MCs neurotoxic potential [33]. *C. elegans* express homologs of human PP1 [38] and 2A [39], and it has previously been shown that *C. elegans* exposed to environmentally relevant concentrations of MC-LR for 48 h exhibit concentration-dependent effects on generation time, brood size, locomotion, lifespan, and body size [40]. A follow-up study demonstrated that 24 h exposure to MC-LR inhibited behaviors mediated by the AWA volatile odor sensory neuron, ASE water-soluble sensory neuron, and the AFD and AIY neurons, which control thermotaxis, and suppressed neuron-specific genes controlling these responses [41]. While these studies suggest that *C. elegans* are sensitive to MCs, inconsistencies regarding systemic toxicity, exposure methods, and behavior analysis, left many questions unanswered. The primary goal of this study was to develop a rigorous and systematic method to use the chemotaxis assay to compare the relative potency of two MC variants, MC-LR and MC-LF, on behaviors mediated by two specific volatile odor sensory neurons, the AWC and AWA. The second goal was to determine whether MC-induced behavioral changes are mediated through the inhibition of PP1 and/or PP2A.

## 2. Results and Discussion

### 2.1. Statistical Evaluation of Chemotaxis Using a Generalized Linear Model

**Figure 1:**
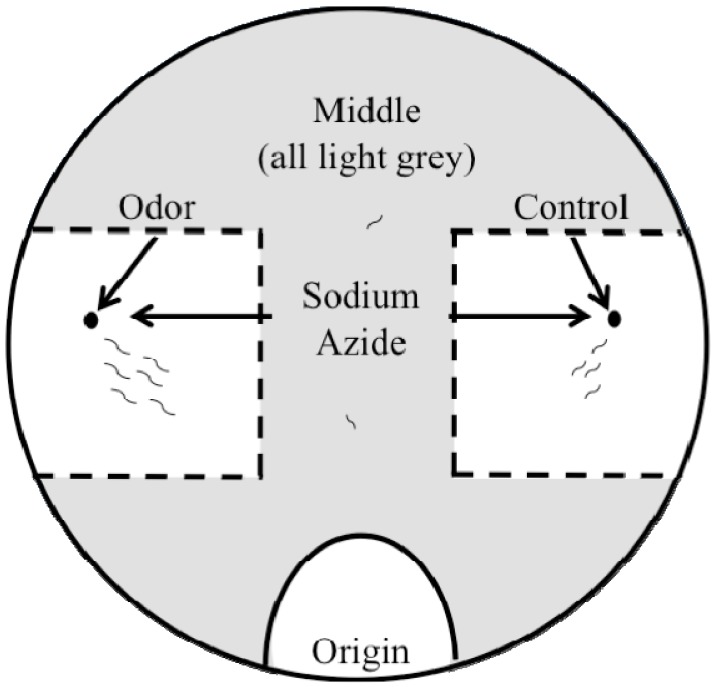
Schematic illustrating the endpoints quantified in the chemotaxis assay. Boxes and point sources (for odor and control) are marked on the assay plate prior to adding assay agar. Sodium azide is placed at the point sources to immobilize worms once they reach the odor or control. Odor and control (ethanol) solutions are added to the respective point sources. Worms are placed at the origin and move to the odor or to the control (white areas with dotted lines) or within the middle region (light grey). Worms are counted in the odor box, in the control box, and in the middle region, whereas worms still at the origin are not included in the analysis.

Three endpoints are typically quantified in the chemotaxis assay: (1) the number of worms that move towards the point source of the odor (benzaldehyde or diacetyl), referred to as the odor; (2) the number of worms that move towards the point source of the odor diluent (ethanol), referred to as the control; and (3) the number of worms that move to the region midway between the odor and the control, referred to as the middle (Figure 1). Typically, the chemotaxis index is used to evaluate changes in chemotactic behavior. The chemotaxis index is a ratio from −1 (100% repelled by an odor) to 1 (100% attracted to an odor) and is calculated as the ((number of worms at the odor)-(number of worms at the control))/(total number of worms). There are two primary concerns in using the chemotaxis index to assess neurotoxicity: (1) ratios bound from −1 to 1 create a dataset that is not normally distributed; and (2) statistical approaches used to compare data sets do not allow negative numbers. Thus, to evaluate the neurotoxicity of MCs using chemotactic response data, we instead developed a generalized linear model using the quasibinomial family.

**Figure 2:**
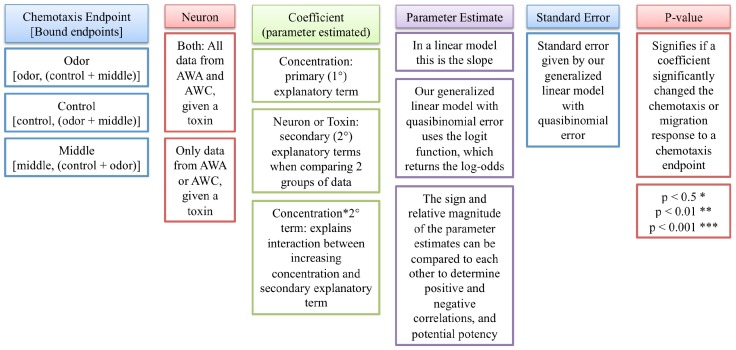
Generalized linear model summary tables. Our generalized linear model characterizes the chemotactic response as a function of MC concentration. The chemotaxis endpoint describes the type of response analyzed (chemotactic response towards odor source point as well as migration responses towards control and middle endpoints). Endpoints were bound according to the type of response analyzed, creating a single object that became the response variable. The neuronal cell type analyzed and the estimated coefficients are additional inputs to the model. Parameter estimates, standard errors and *p*-values are calculated by the generalized linear model.

A generalized linear model using the binomial family takes into account the proportional properties of chemotactic response data: the strictly bound data, non-constant variance and non-normal errors. Due to overdispersion in the data, the quasibinomial family was used in our model, with the consequence of larger standard errors and more conservative *p*-values. Our model required the comparison of two outputs; therefore, an individual endpoint of the chemotaxis assay (odor, control or middle) was compared to the other two endpoints added together. This resulted in three different ways to analyze the chemotaxis data based on the endpoint of interest (Figure 2). To analyze the chemotactic response to an odor, the number of worms at an odor was compared to the rest of the sample. To analyze alternative patterns of movement for worms that did not move towards the odor, the control or middle worms were compared to the rest of the sample. The two outputs needed from each individual chemotaxis assay were matched by binding them together, creating a single object that became the response variable. All chemotaxis assays for a given toxin could then be grouped and analyzed.

The data are reported in boxplot graphs and summary tables. The graphs illustrate trends in the chemotactic response on the y-axis, calculated as the proportion of worms moving towards benzaldehyde (to test AWC function) or towards diacetyl (to test AWA function), as a function of increasing MC concentration. A proportion of 0.5 on the y-axis represents a neuron failing to detect an odorant, since equal number of worms did and did not go to the odor. Summary tables describe the input (chemotaxis endpoint, neuron type, and estimated coefficients) used for the generalized linear model, and report parameter estimates as calculated using the generalized linear model, with standard error and p-values (Figure 2), with interaction terms listed if significant. Since the generalized linear model uses a link function (called logit) to transform the chemotactic response data, the parameter estimates cannot be interpreted directly as a slope. However, the sign and relative sizes of the parameter estimates can be used to determine increased or decreased neuron function after toxin exposure, and whether one toxin was more potent than the other.

All models comparing toxin concentration effect on the AWC and AWA neurons determined the neuron coefficient to be significant, demonstrating that the tested *C. elegans* had different chemotactic responses towards each odor, regardless of whether a toxin altered the chemotactic response with increasing concentration. This is in agreement with the fact that AWC and AWA act as independent neurons at low concentrations of odor (1:1000 for diacetyl). *C. elegans* in which AWA sensory neurons were killed with a laser beam are able to detect diacetyl at high concentrations of odor (1:10 for diacetyl) due to cellular redundancy between olfactory neurons at high odorant concentrations [37]. The odorant concentration used in this study (1:1000 for diacetyl), combined with our generalized linear models describing two separate sensory neuron responses to the two odors tested, suggests that changes in detection of diacetyl are likely a result of altered AWA function.

To the best of our knowledge, this is the first time a generalized linear model has been used to analyze chemotaxis data. This analytical approach provides a robust method for analyzing *C. elegans* chemotaxis behavior after exposure to a range of MC concentrations, and allows for comparing grouped data to determine differences between neuron-specific functions as a function of MC concentration.

### 2.2. Outlier Chemotaxis Assays

Grubbs’ test was used to identify potential outlier control assays. One of over a hundred control assays was identified as a potential outlier, and further investigation found worms had the same chemotactic response to benzaldehyde and control during this assay. Benzaldehyde can crystallize, and this can change the odor from an attractant to a neutral or repellant odor. We assumed the benzaldehyde was crystallized for this assay, as a new vial of benzaldehyde was used for the next assay and controls were normal. Therefore, the control assay was discarded, along with all exposure assays associated with that particular control assay. To avoid bias for potential trends in the data and to account for any possible error and variation, all other data points were used.

### 2.3. MC-LR Impairs AWA Function, but not AWC Function

**Figure 3:**
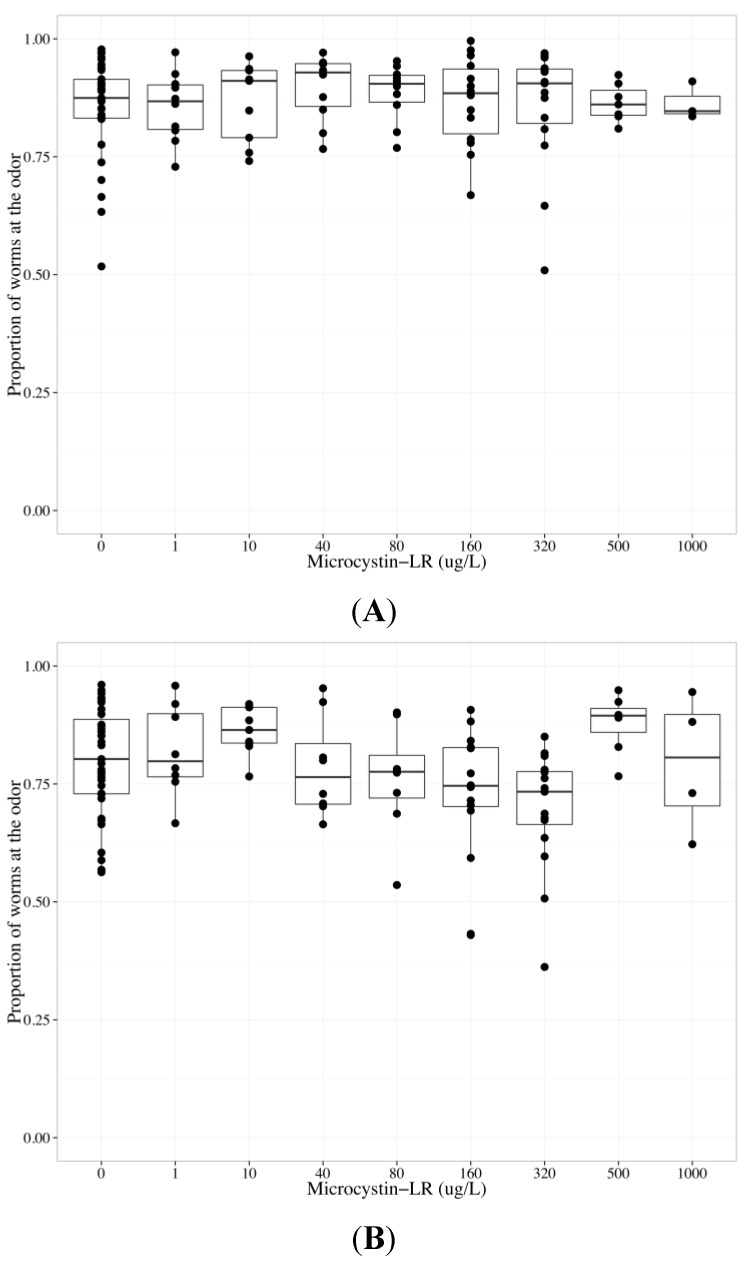
The chemotactic response of wildtype *C. elegans* to benzaldehyde (AWC-mediated chemotaxis) or diacetyl (AWA-mediated chemotaxis) after exposure to 0–1000 µg/L microcystin-LR (MC-LR) for 24 h. The bold horizontal bar in the middle of the box is the median value, the bottom and top of the box represent the 25th and 75th percentiles, respectively, and whiskers extend to the farthest data point within 1.5 interquartile ranges from the edges of the box, with extreme values separated as circles. N ≥ 6 chemotaxis assays (except 1000 µg/L, *n* ≥ 3), with 100–300 worms used per assay. The chemotactic response is the proportion of worms at the odor compared to the total number of worms analyzed in the assay, and 0.5 represents no detection of odor. (**A**) MC-LR did not change the chemotactic response to benzaldehyde, suggesting that MC-LR does not impair AWC function; (**B**) Chemotaxis towards diacetyl diminished as MC-LR concentrations increased up to 320 µg/L; at higher MC-LR concentrations, chemotactic responses to diacetyl were either increased (500 µg/L MC-LR) or no different (1000 µg/L MC-LR) from control.

To determine whether MC-LR altered AWC and/or AWA function, we analyzed chemotaxis towards benzaldehyde, which is detected by AWC sensory neurons, *versus* diacetyl, which is detected by AWA sensory neurons, in wildtype worms exposed to MC-LR from 0 to 1000 µg/L (final agar concentrations). Because we observed a non-monotonic (inverted concentration-relationship) chemotactic response to diacetyl, with a decreasing chemotactic response observed at MC-LR concentrations up to but not above 320 µg/L, data collected from worms exposed to MC-LR at concentrations ≤320 µg/L were analyzed separately from data collected from worms exposed to >320 µg/L MC-LR (Figure 3). Increasing MC-LR concentration diminished the chemotactic response to odors at concentrations ≤320 µg/L (*p* < 0.001); however, there was a statistically significant difference between AWC and AWA neurons (*p* < 0.01), and there was a significant interaction term between MC-LR concentration and neuron type (*p* < 0.05) (Table 1). To investigate the difference between AWC- and AWA-mediated chemotaxis after MC-LR exposure, neuron-specific data were analyzed separately. There was no effect of MC-LR on chemotaxis towards benzaldehyde (Table 1, Figure 3a). MC-LR significantly decreased chemotactic response to diacetyl in a concentration-dependent manner (*p* < 0.001, Table 1, Figure 3b). Worms that could not sense diacetyl went to both the control and middle with increased MC-LR concentration exposure (*p* < 0.01 for each endpoint, Table 1).

**Table 1:** Behavior of adult wildtype worms exposed to 0–320 µg/L microcystin-LR (MC-LR) for 24 h. Increasing MC-LR concentration diminished the chemotactic response to an odor (significant concentration coefficient), AWC *versus* AWA mediated chemotaxis were different (significant neuron coefficient) and the AWC and AWA-mediated chemotaxis changed differently with increasing MC-LR concentration (significant concentration*neuron interaction). Independent analyses of the behaviors mediated by the two neuronal cell types indicate that MC-LR impaired AWA function, but not AWC function.

Chemotaxis endpoints	Neuron	Coefficient	Parameter estimate	Standard error	*p*-value
Odor	Both	Concentration	−0.00190	0.000543	0.000571 ***
	Both	Neuron	0.433	0.138	0.00200 **
	Both	Concentration*Neuron	0.00180	0.000858	0.0370 *
	AWC	Concentration	−0.000101	0.000682	0.883
	AWA	Concentration	−0.00190	0.000528	0.000501 ***
Middle	AWC	Concentration	0.0000272	0.000604	0.964
	AWA	Concentration	0.00170	0.000570	0.00356 **
Control	AWC	Concentration	0.000139	0.000851	0.871
	AWA	Concentration	0.00161	0.000561	0.00498 **

Our results indicated that MC-LR impaired the function of the AWA sensory neuron, but not the AWC sensory neuron. Worms exposed to MC-LR were capable of moving and exhibited appropriate AWC-mediated chemotaxis, suggesting that muscle function, coordination, and energy needed for chemotaxis were not impaired. Previous studies do not sufficiently separate potential neurotoxicity from systemic toxicity. One study exposed larval 4 (L4) worms to MC-LR for 2 days at concentrations up to 160 µg/L, without food. Lifespan, body size, brood size, and locomotion behavior functions decreased, while generation time and stress responses in the intestine, nervous system and vulva (using green fluorescent protein labeled heat shock promoter *hsp-16-2*) increased with increasing concentrations of MC-LR [40]. Only the stress response in muscle tissue was not affected by MC-LR. As MC-LR targets PP1 and 2A, 24 h exposure to MC-LR may target cells other than the AWA sensory neurons. In our study, AWC-mediated chemotaxis remained constant with increasing concentrations of MC-LR. Therefore changes in the AWA-mediated chemotactic response after MC-LR exposure are likely a result of impaired AWA function. Studies using locomotion behavior as an endpoint after metal exposure found younger stages of *C. elegans* larva (L1-L4) to be more susceptible than young adults [42], with metal sensitivity decreasing with age. Therefore, our exposure, using adult worms, for 24 h in the presence of food may prevent some systemic toxicity by MC-LR.

A follow up study found L4 worms exposed to MC-LR for 24 h at concentrations up to 160 µg/L with food had impaired volatile odor (AWA), water-soluble odor (ASE), and temperature (AFD) sensory neuron and interneuron (AIY) function [41]. The chemotactic responses to odors were analyzed using the percent change in the chemotaxis index as the endpoint, and one-way analysis of variance (ANOVA) followed by a Dunnett’s *t*-test was used to determine significant differences between chemotaxis indexes at each MC-LR concentration. As discussed in our paper, using the chemotaxis index as an endpoint may not be a rigorous enough statistical approach to determine whether increasing concentrations of MC-LR impact specific neurons. This paper was inconsistent with the analysis of separating systemic toxicity from neurotoxicity: the authors concluded that exposure to MC-LR concentrations ≥40 µg/L significantly decreased AWA function, yet only investigated changes in mechanotransduction and moving velocity after exposure to MC-LR concentrations <40 µg/L. No changes in mechanotransduction or moving velocity were observed after exposure to MC-LR concentrations <40 µg/L, which does not rule out changes in mechanotransduction or moving velocity after exposure to MC-LR concentrations ≥40 µg/L. Furthermore, as locomotion behaviors (head thrash and body bends) were negatively impacted at 10 and 40 µg/L MC-LR (L4 and 48 h exposure) [40], it is possible that L4s exposed to MC-LR for 24 h also had impaired locomotion behavior. Our results, using a generalized linear model, agree with the previous data that MC-LR impairs the AWA neuron. It is possible that neurons other than the AWA, such as the ASE, AFD and AIY as mentioned above, may be targeted by MC-LR. Downstream neurons associated with the AWA may be impaired by MC-LR, resulting in the observed decrease in chemotactic response towards diacetyl. The lack of AWC-mediated changes suggests that the MC-LR induced AWA neurotoxicity is a specific impact on the AWA or associated neurons, rather than a systemic response. Systemic toxicity would most likely impact all chemotactic behaviors. The lack of AWC-mediated changes also suggests that downstream interneurons required for both AWA and AWC function are not targeted by MC-LR, as impairment would most likely result in altered AWA- and AWC-mediated behaviors.

MC-LR may have altered AWA sensory neuron function in a concentration-dependent non-monotonic response via inhibition of PP1/PP2A at concentrations at or below 320 µg/L, while concentrations above 320 µg/L may have enhanced PP expression. Although inhibition of PP1 and 2A is the most documented mechanism of action for MCs, increased expression of PP2Ac [43] and PP2Cα2 [44], and increased overall protein phosphatase activity have been observed after non-lethal exposures in fish [44,45]. MCs can induce intracellular calcium influx *in vitro* [45,46,47], yet the mechanisms by which MCs induce calcium changes, is not fully understood. Phosphorylation state and calcium regulation have been linked to potentially opposing effects: drosophila transient receptor potential (TRP) and TRP-like calcium channels require dephosphorylation for activation, while activation of L-type calcium channels in cardiac cells requires phosphorylation by protein kinase A and C [48,49]. MC-LR may alter AWA function by changing cellular phosphorylation states and disrupting intracellular calcium levels because AWA sensory neurons rely on controlled intracellular calcium levels through the TRP-like channel OSM-9 coupled with a G-protein coupled receptor cascade [50,51] and L-type calcium channels [52]. It is possible that, at high MC-LR concentrations, toxin removal from neurons was reduced, resulting in inhibition of the induced PPs and a cellular phosphorylation state similar to lower concentrations of MCs.

In our studies the AWC sensory neurons did not appear to have altered function after exposure to increasing concentrations of MC-LR. Although AWC sensory neuron relies on calcium signaling for the detection of benzaldehyde [52], the primary signaling cascade requires a cyclic nucleotide gated channel [53]. Olfactory adaptation, a process in which *C. elegans* become desensitized to odor stimulus, requires OSM-9, but is not required to detect benzaldehyde [50]. Therefore, MC-induced calcium deregulation may only impair detection of diacetyl due to the differences in AWA and AWC olfactory transduction pathways. As previously mentioned, MCs may also target interneurons specific to AWA neuron function [37,54].

### 2.4. MC-LF Impairs AWA Function, but not AWC Function

**Figure 4:**
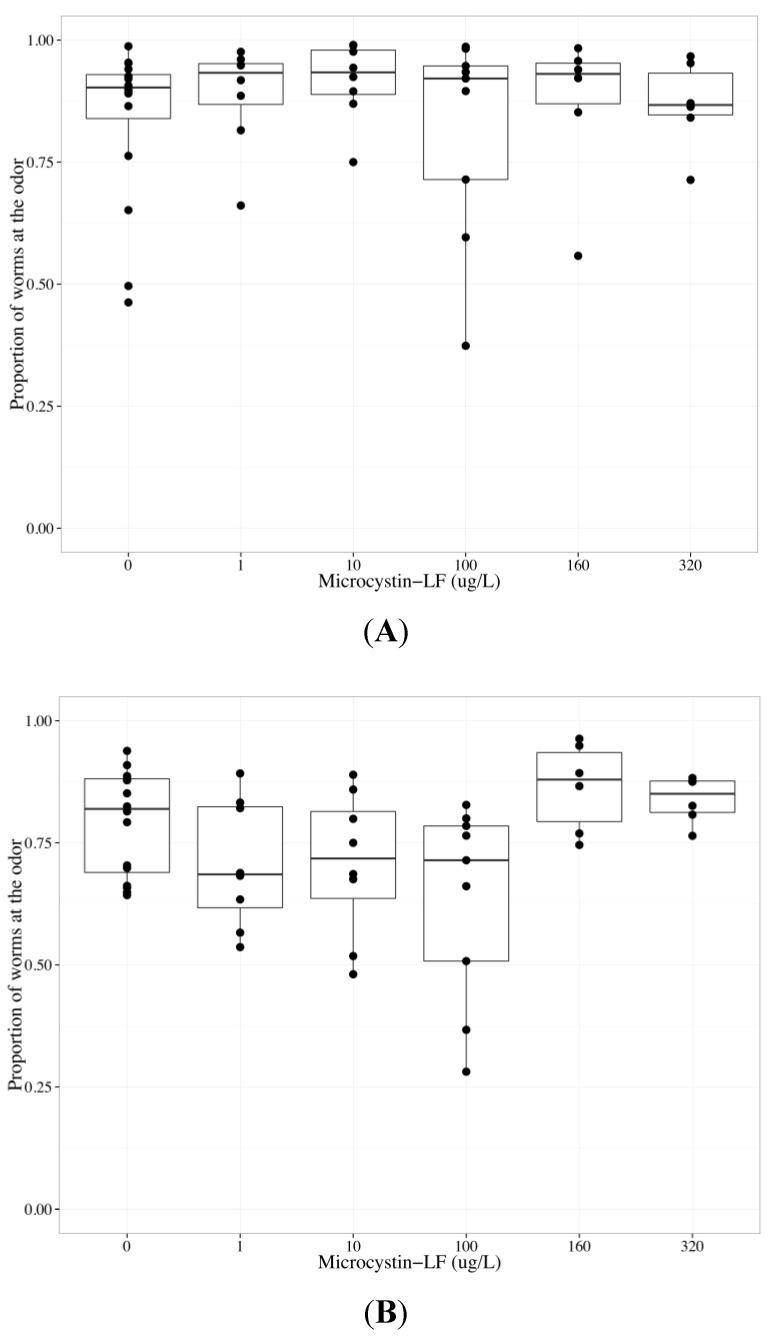
Chemotaxis of wildtype *C. elegans* towards benzaldehyde (AWC-mediated chemotaxis) or diacetyl (AWA-mediated chemotaxis) after exposure to 0–320 µg/L microcystin-LF (MC-LF) for 24 h. The bold horizontal bar in the middle of the box is the median value, the bottom and top of the box represent the 25th and 75th percentiles, respectively, and whiskers extend to the farthest data point within 1.5 interquartile ranges from the edges of the box, with extreme values separated as circles. N ≥ 6 chemotaxis assays with 100–300 worms used per assay. The chemotactic response is the proportion of worms at the odor compared to the total number of worms analyzed in the assay and 0.5 represents no detection of odor. (**A**) MC-LF did not change the chemotactic response to benzaldehyde, suggesting that MC-LF does not impair AWC function; (**B**) Chemotaxis towards diacetyl diminished as MC-LF concentrations increased up to 100 µg/L; at higher MC-LF concentrations, chemotactic responses to diacetyl were either increased (160 µg/L MC-LF) or no different (320 µg/L MC-LF) from control.

MC-LF is less studied than MC-LR, yet this more hydrophobic MC variant has been found in the environment [55,56] and is reported to be more toxic to neurons and intestinal cells than MC-LR [26,27,57]. Similar to MC-LR, chemotactic responses to diacetyl exhibited a non-monotonic concentration-effect relationship, with a decreasing chemotactic response observed at MC-LF concentrations up to but not beyond 100 µg/L. Therefore, data collected from worms exposed to MC-LF at concentrations ≤100 µg/L were analyzed separately from data collected from worms exposed to >100 µg/L MC-LF (Figure 4). Increasing MC-LF concentration decreased chemotaxis to odors (*p* < 0.1) with statistically significant differences between AWC- *versus* AWA-mediated chemotaxis (*p* < 0.001) (Table 2). To investigate the difference between AWC- and AWA-mediated chemotaxis after MC-LF exposure, neuron-specific data were analyzed separately. MC-LF did not change the chemotactic response to benzaldehyde (Table 2, Figure 4a), but did significantly inhibit chemotaxis towards diacetyl (*p* < 0.05, Table 2, Figure 4b). The concentration coefficient in the first MC-LF model, which incorporated both neuronal cell types, was not significant since the negative effect of concentration on AWA-mediated chemotaxis was not enough to skew the combined data, which is in contrast to the MC-LR data. The low p-value for the neuron coefficient signified a possible difference between the neurons. Worms that could not sense diacetyl migrated to the middle region with increasing MC-LF exposure (*p* < 0.001, Table 2).

Our results indicate MC-LF altered AWA-mediated chemotaxis but not AWC-mediated chemotaxis, similar to MC-LR. As MC-LR and MC-LF covalently bind and alter PP1 and 2A function similarly, MC-LF may alter AWA function with the same mechanism of action previously proposed for MC-LR.

**Table 2:** Behavior of adult wildtype worms exposed to 0–100 µg/L microcystin-LF (MC-LF) for 24 h. AWC- and AWA-mediated chemotactic responses were different (significant neuron coefficient). Independent analyses of the behaviors mediated by the two neuronal cell types indicated that MC-LF impaired AWA function, but not AWC function.

Chemotaxis endpoint	Neuron	Coefficient	Parameter estimate	Standard error	*p*-value
Odor	Both	Concentration	−0.00593	0.00342	0.0873
	Both	Neuron	0.970	0.223	4.04 × 10^−5^ ***
	AWC	Concentration	−0.00216	0.00460	0.641
	AWA	Concentration	−0.00593	0.00280	0.0403 *
Middle	AWC	Concentration	0.000643	0.00291	0.826
	AWA	Concentration	0.00714	0.00197	0.00082 ***
Control	AWC	Concentration	0.00267	0.00551	0.631
	AWA	Concentration	0.00375	0.00327	0.259

### 2.5. MC-LF may be More Potent than MC-LR at Impairing AWA Function

To determine the relative potency of MC-LR and MC-LF neurotoxic effects on AWA sensory neuron function, data from concentrations of each MC that resulted in decreased chemotactic response to diacetyl (up to 320 µg/L for MC-LR, 100 µg/L for MC-LF) were used. With increasing MC concentration, chemotaxis towards diacetyl diminished (*p* < 0.001), and MC-LR and MC-LF impaired AWA function differently (*p* < 0.01) as determined by the significant toxin coefficient (Table 3). The negative parameter estimate for MC-LF, −0.00593 (Table 2), was more negative than the parameter estimate for MC-LR, −0.00190 (Table 1), suggesting MC-LF to be more potent than MC-LR at impairing AWA function. This conclusion is in agreement with recent data showing MC-LF to be more potent than MC-LR *in vitro* [56], with respect to cytotoxicity, PP activity and tau phosphorylation, neurite length, and cell proliferation and morphology.

**Table 3:** AWA-mediated chemotaxis of adult wildtype worms exposed to 0–320 µg/L microcystin-LR (MC-LR) or 0–100 µg/L microcystin-LF (MC-LF) analyzed using the generalized linear model. Increasing MC concentration inhibited the chemotactic response to diacetyl (significant concentration coefficient), and MC-LR and MC-LF differentially impaired AWA function (significant toxin coefficient). MC-LF has a larger negative parameter estimate than MC-LR, suggesting MC-LF is more potent than MC-LR.

Chemotaxis endpoint	Toxin	Coefficient	Parameter estimate	Standard error	*p*-value
Odor	Both	Concentration	−0.00204	0.000524	0.000152 ***
	Both	Toxin	0.381	0.141	0.00763 **

The hydrophobic properties of MC-LF could facilitate and increase cellular uptake, causing a more rapid decrease in AWA function with increasing concentration. Also, OATPs have differential specificity for specific MC variants [16,26,27], suggesting OATP isoforms concentrated in different cell types may facilitate uptake of specific MC congeners. This might explain why MC-LR is considered a more potent hepatotoxin, but MC-LF is a more potent neurotoxin. MC-LF’s potency may cause the worms to bypass both diacetyl and control endpoints, and thus go straight forward during the chemotaxis assay. This could explain our observation that worms went to the middle endpoint when unable to sense diacetyl.

### 2.6. Tautomycin Does not Impair AWC or AWA function, While Okadaic Acid Impairs Both

**Figure 5:**
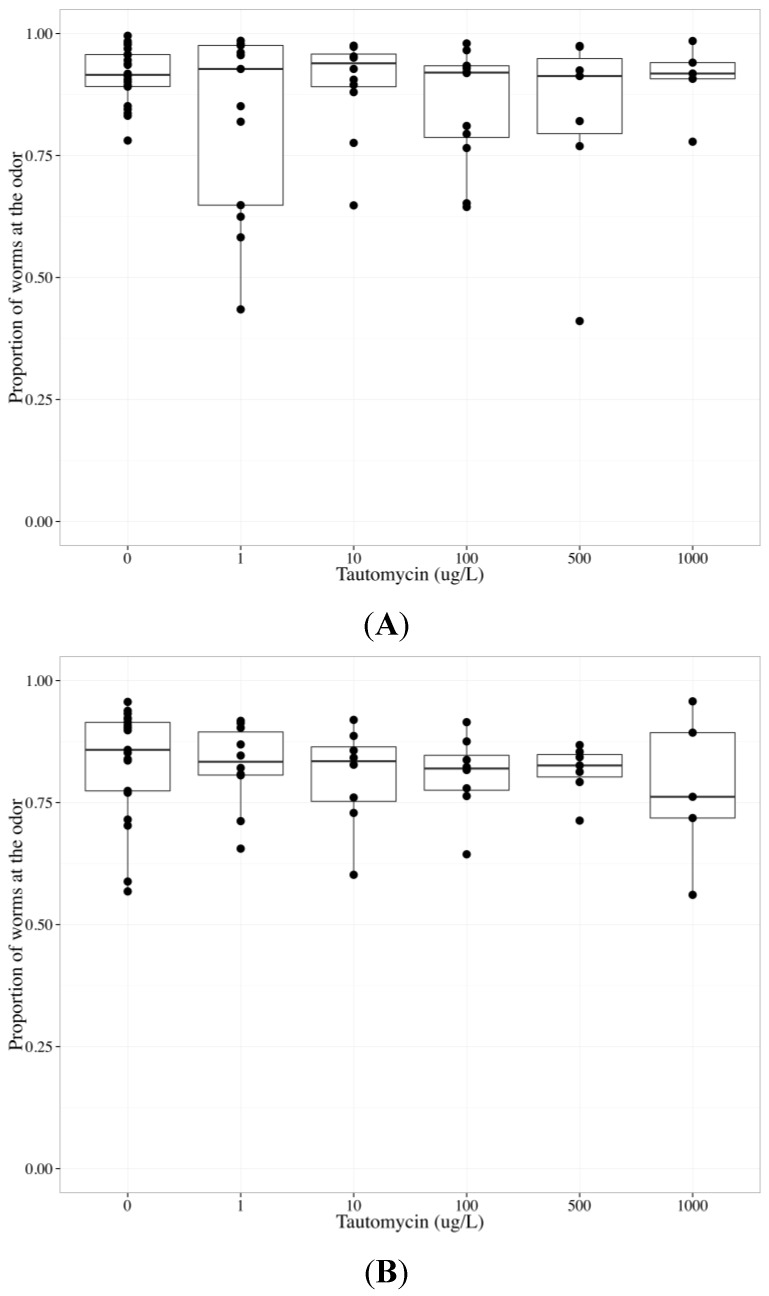
The chemotactic response of wildtype *C. elegans* to benzaldehyde (AWC-mediated chemotaxis) or diacetyl (AWA-mediated chemotaxis) after exposure to 0–1000 µg/L tautomycin for 24 h. The bold horizontal bar in the middle of the box is the median value, the bottom and top of the box represent the 25th and 75th percentiles, respectively, and whiskers extend to the farthest data point within 1.5 interquartile ranges from the edges of the box, with extreme values separated as circles. N ≥ 6 chemotaxis assays with 100–300 worms used per assay. The chemotactic response is the proportion of worms at the odor compared to the total number of worms analyzed in the assay and 0.5 represents no detection of odor. Tautomycin did not change the chemotactic response to benzaldehyde (**A**); or diacetyl (**B**).

**Figure 6:**
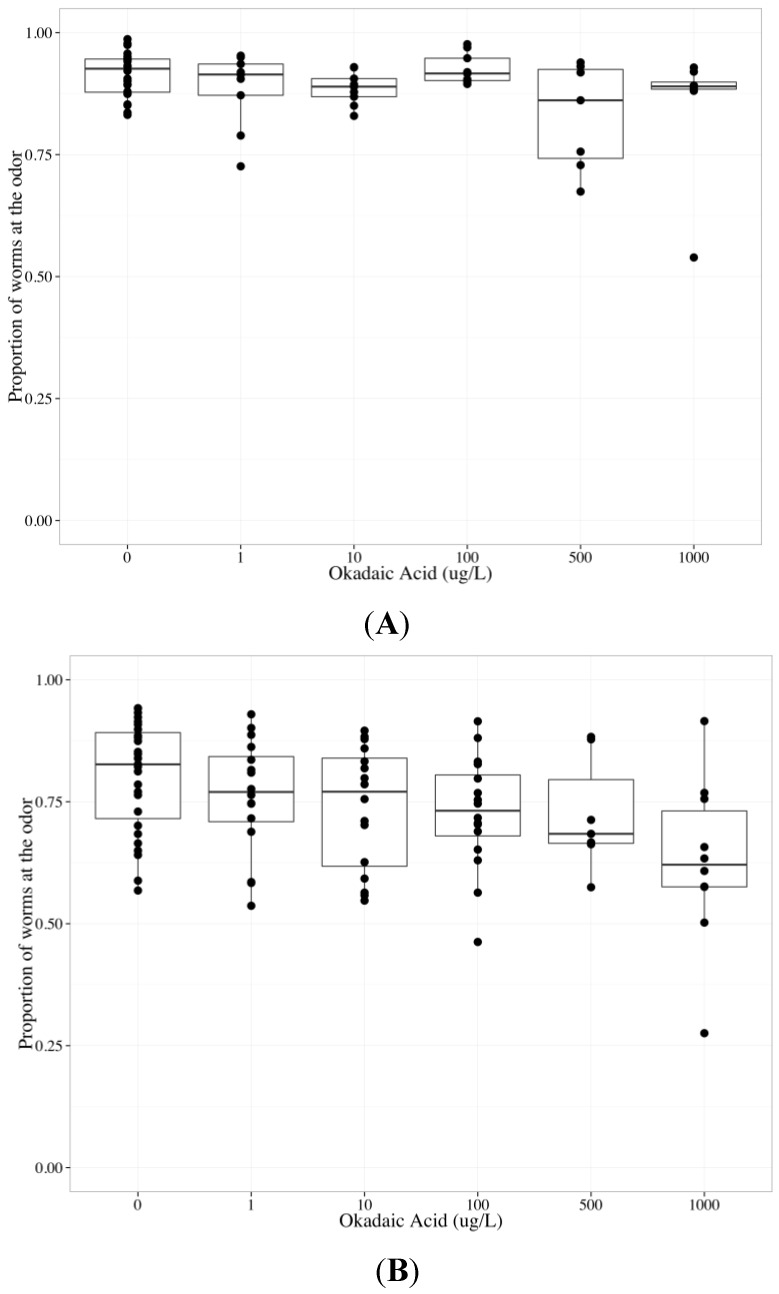
The chemotactic response of wildtype *C. elegans* to benzaldehyde (AWC-mediated chemotaxis) or diacetyl (AWA-mediated chemotaxis) after exposure to 0–1000 µg/L okadaic acid for 24 h. The bold horizontal bar in the middle of the box is the median value, the bottom and top of the box represent the 25th and 75th percentiles, respectively, and whiskers extend to the farthest data point within 1.5 interquartile ranges from the edges of the box, with extreme values separated as circles. N ≥ 6 chemotaxis assays with 100–300 worms used per assay. The chemotactic response is the proportion of worms at the odor compared to the total number of worms analyzed in the assay and 0.5 represents no detection of odor. Increasing okadaic acid concentration diminished the chemotactic response to (**A**) benzaldehyde and (**B**) diacetyl.

MC-LR is a very potent inhibitor of PP1 and PP2A (inhibitory constant (K_i_) = 0.04 nM and 0.01 nM, respectively), while tautomycin inhibits PP1 more potently than PP2A (K_i_ = 0.43 nM and 340 nM, respectively) and okadaic acid inhibits PP2A more potently than PP1 (K_i_ = 0.03 nM and 147 nM, respectively). The K_i_ of MC-LR, tautomycin and okadaic acid were previously measured using purified rabbit muscle PP1 and 2A and p-nitrophenyl phosphate [58]. To determine whether tautomycin altered AWC and/or AWA function, we analyzed data collected from wildtype worms exposed to tautomycin from 0 to 1000 µg/L (final agar concentrations). Increasing tautomycin concentration did not alter the chemotactic response to the odors; however, there was a statistically significant difference between AWC and AWA neurons (*p* < 0.05, Table 4). There was no effect of tautomycin on chemotaxis towards benzaldehyde (Table 4, Figure 5a) or diacetyl (Table 4, Figure 5b). To determine whether okadaic acid altered AWC and/or AWA function, we analyzed data collected from wildtype worms exposed to okadaic acid from 0 to 1000 µg/L (final agar concentrations). Increasing okadaic acid concentration diminished the chemotactic response to odors (*p* < 0.01) and there was a statistically significant difference between AWC and AWA neurons (*p* < 0.001) (Table 5). To investigate the difference between AWC- and AWA-mediated chemotaxis after okadaic acid exposure, neuron-specific data were analyzed separately. Okadaic acid significantly decreased chemotaxis towards benzaldehyde (*p* < 0.05, Table 5, Figure 6a) and diacetyl (*p* < 0.05, Table 5, Figure 6b) in a concentration-dependent manner. Populations that could not sense the odors went to the middle with increasing okadaic acid concentration exposure (*p* < 0.001, each neuron, Table 5).

**Table 4:** Behavior of adult wildtype worms exposed to 0–1000 µg/L tautomycin for 24 h. Increasing tautomycin concentration did not change chemotactic response to odors, and AWC- *versus* AWA-mediated chemotactic responses to odors were different (significant neuron coefficient). Independent analyses of the behaviors mediated by the two neuronal cell types indicated that tautomycin did not impair AWA or AWC function.

Chemotaxis endpoint	Neuron	Coefficient	Parameter estimate	Standard error	*p*-value
Odor	Both	Concentration	−0.000206	0.000231	0.375
	Both	Neuron	0.413	0.165	0.0136 *
	AWC	Concentration	−3.14 × 10^−5^	4.23 × 10^−4^	0.941
	AWA	Concentration	−0.000343	0.000226	0.134
Middle	AWC	Concentration	0.000313	0.000353	0.378
	AWA	Concentration	0.000182	0.000194	0.353
Control	AWC	Concentration	−0.000127	0.000488	0.796
	AWA	Concentration	0.000371	0.000279	0.188

**Table 5:** Behavior of adult wildtype worms exposed to 0–1000 µg/L okadaic acid for 24 h. Increasing okadaic acid concentration diminished the chemotactic response to odors (significant concentration coefficient), and AWC- *versus* AWA-mediated chemotactic responses to odors were different (significant neuron coefficient). Independent analyses of the behaviors mediated by the two neuronal cell types indicated that okadaic acid impairs AWA and AWC function.

Chemotaxis endpoint	Neuron	Coefficient	Parameter estimate	Standard error	p-value
Odor	Both	Concentration	−0.000442	0.000137	0.00148 **
	Both	Neuron	1.03	0.120	6.57 × 10^−15^ ***
	AWC	Concentration	−0.000475	0.000188	0.0141 *
	AWA	Concentration	−0.000424	0.000189	0.0274 *
Middle	AWC	Concentration	0.000625	0.000144	4.89 × 10^−5^ ***
	AWA	Concentration	0.000674	0.000144	1.06 × 10^−5^ ***
Control	AWC	Concentration	0.000282	0.000271	0.303
	AWA	Concentration	0.000145	0.000236	0.54

A small hydrophobic molecule, tautomycin is thought to readily permeate cells, though like MCs and okadaic acid, its ability to cross adult *C. elegans* strong cuticle is unknown [59]. At the concentrations we tested, *C. elegans* behavior did not change with exposure to tautomycin, and the AWC and AWA sensory neurons remained functional. Possible explanations for the lack of change in chemotactic response to odors include: (1) tautomycin did not reach the target site; (2) PP1 is not critical for *C. elegans* sensory neuron function; or (3) if PP1 is critical for sensory neuron function, alternative signaling pathways were initiated when tautomycin inhibited PP1. Both AWC- and AWA-mediated chemotaxis decreased after okadaic acid exposure. PP2A may be required for AWC and AWA sensory neuron function, or okadaic acid may have caused systemic toxicity and inhibited PP2A in muscle cells. Our findings that okadaic acid impaired both AWC and AWA function, while tautomycin did not alter the function of either neuron, suggest that MCs do not impair AWA function through PP1 or 2A inhibition. Potential differences between the inhibitory constants of *C. elegans* and mammalian PP1 and 2A for MCs, okadaic acid and tautomycin may explain why neither okadaic acid nor tautomycin altered behavior similar to MCs.

MCs inhibit a third class of PPs, the calcium/calmodulin-dependent PP2B, although with 1000-fold lower potency than their inhibition of PP1 and 2A [60]. As previously mentioned, MCs have been reported to alter intracellular calcium levels [45,46,47]; since calcium/calmodulin-dependent protein kinase II is required for MC-induced apoptosis [61,62], changes in calcium levels may contribute to the effects of MCs on PP2B activity. Interestingly, the *tax-6 C. elegans* mutant, which contains defective PP2B enzymes, mimics many of the phenotypes observed in wildtype *C. elegans* exposed to MC-LR exposure [63,64]. Changes in calcium-dependent events that lead to inverted concentration-related effects have been demonstrated before. Mice developmentally exposed to the neurotoxins polychlorinated biphenyls had altered behavior and dendritic morphology at low doses, yet were similar to controls at high doses, resulting in non-monotonic responses [65]. Therefore, MCs may cause an inverse concentration-relationship with AWA-mediated chemotaxis via disruption of PP2B function by altered calcium regulation. The mechanism of action by which MCs alter calcium may be specific to the AWA, compared to the AWC, due to differences in required calcium channels for each sensory neuron, as previously mentioned.

## 3. Experimental Section

### 3.1. Strains

The wild-type *C. elegans* Bristol strain (N2) was purchased from the *Caenorhabditis elegans* Genetic Center, University of Minnesota. The strain was maintained on nematode growth medium (NGM) plates seeded with *Escherichia coli* strain OP50 and incubated at 20 °C according to standard protocol [31]. Populations of synchronized worms were obtained by transferring two larval stage 4 (L4) nematodes to a seeded small NGM plate (60 mm, 10 mL NGM agar) and after 5 days adult progeny were collected and washed.

### 3.2. Materials

M9 buffer, S. Basal buffer and NGM plates were purchased from IPM Scientific (Eldersburg, MD, USA). Purified agar-agar for behavioral assays was purchased from EMD Millipore (Billerica, MA, USA). All chemicals and odorants were purchased from Sigma-Aldrich (St. Louis, MO, USA) at analytical grade or higher.

### 3.3. Protein Phosphatase Inhibitors

MC-LR was purchased from Sigma-Aldrich (St. Louis, MO, USA, discontinued) and MC-LR and MC-LF were purchased from Enzo Life Sciences (Farmingdale, NY, USA) and had purity of ≥95% (HPLC). Okadaic acid was purchased from LC Laboratories (Woburn, MA, USA, purity 98%). Tautomycin was purchased from AG Scientific, Inc. (San Diego, CA, USA, discontinued, purity 97%) and Wako Chemicals (Richmond, VA, purity ≥ 90%). MC-LR (1 mg), MC-LF (100 µg), okadaic acid (100 µg) and tautomycin (100 µg) standards were dissolved in 1 mL 2.5% methanol and stored at −20°C.

### 3.4. 24-Hour Inhibitor Exposure

Synchronized populations of young adults were washed three times in Eppendorf tubes using S. Basal buffer, and after worms settled, the supernatant was completely removed. Since toxin standards were dissolved in methanol and diluted in water, control exposures were based on the methanol concentration in the highest toxin concentration tested, and never went above 0.025% final agar concentration. Pilot studies demonstrated exposure up to 1% methanol (final agar concentration) resulted in no change in benzaldehyde or diacetyl chemotaxis (*n* = 4, unpublished work). Freshly prepared exposure solution concentrations of methanol, MC-LR, MC-LF, okadaic acid, and tautomycin were calculated based on the final volume of 10 mL agar in each NGM plate. One hundred microliters of exposure solution (diluted with water) was added to each Eppendorf pellet of worms, and worms with solution were transferred to a fresh seeded NGM plate and kept at 20 °C for 24 h. Therefore, the calculated exposure concentrations were based on one hundred microliters of concentrated control or toxin diffusing in 10 mL agar to the listed final concentrations. Worms were exposed to 1, 10, 40, 80, 160, 320, 500, and 1000 µg/L MC-LR, 1, 10, 100, 160 and 320 µg/L MC-LF, and 1, 10, 100, 500, and 1000 µg/L okadaic acid and tautomycin. Initial studies included MC-LR concentrations based on previously published work [41] and expanded to include a larger range of doses as additional toxins were added to our experimental approach.

### 3.5. Chemotaxis Assay

After the 24-hour exposure to MCs or selective PP inhibitors, worms were washed twice with S. Basal buffer, and once with water. Water osmolarity may help stimulate the worms to move away from the origin (Noelle L’Etoile, personal communication). Chemotaxis assays were performed on assay agar (1.6% agar, 1 mM MgSO4, 1 mM MgCl2, 5 mM phosphate buffer (pH 6.0)) in 100 mm petri dishes. The odorants benzaldehyde (1:200) and diacetyl (1:1,000) were diluted in ethanol because worms are not attracted to ethanol [34,66]. One microliter of 1M sodium azide was placed at the control and odor point source to immobilize worms, followed by one microliter of diluted odor or control (ethanol) (Figure 1). The rate at which worms migrate to either sodium azide region should be the same, if no odor was present [34]. Preliminary studies in our laboratory demonstrated that with no odor present and ethanol at both point sources, wildtype worms exposed to water for 24 h went to each point source equally (n = 4). The proportion of worms that went to the right point source was 0.349 ± 0.031 standard error (SE), to the left point source was 0.368 ± 0.034 SE, and the middle was 0.282 ± 0.018 SE. Adult worms were placed at the origin, which was equidistant from both control and odor points, on the edge of the plate. Chemotaxis assays were initiated by wicking the water from the worms. After 2 h at 20 °C and overnight at 4 °C the distribution of worms at the odor, control, and middle (grey area, Figure 1) were counted [67].

### 3.6. Statistics

Chemotaxis assays were performed at least six times, except for 1000 µg/L MC-LR exposure, which was performed at least 3 times, on different days with different samples of worms. Samples ranged from 100 to 300 worms per assay. Generally, diacetyl and benzaldehyde chemotaxis assays would be performed at the same time, splitting the washed exposed worms between the two assays. The only outliers considered were outliers in control groups, and the associated results from those outliers were eliminated. To avoid bias for potential trends in the data and to account for any possible error and variation, all other data points were used. Potential outliers were determined using Grubbs’ test (©2013 GraphPad Software, Inc., La Jolla, CA, USA, alpha < 0.05) and if there was a biological or experimental reason to explain the outlier, the outlier was discarded along with associated exposure assays.

To determine if the AWA and/or AWC sensory neurons were altered with increasing concentrations of toxins, a generalized linear model using the quasibionomial family was used. The quasibionomial family was used to account for overdispersion (large residual deviance) from the natural variability in behavior analysis (R program [68]). For our generalized linear model, a chemotaxis endpoint (number of worms at the odor, control or middle) was compared, through a process in the R program called binding, to the other two endpoints added together. For example, the number of worms at the odor was bound to the number of worms at the control and the middle, for a given assay. The bound set of data created by this process became the response variable and the concentration was the explanatory variable.

When developing our statistical method, we found that analyzing the chemotactic response after exposure to MC-LR up to 320 µg/L using only two chemotaxis endpoints (odor and control) resulted in a similar outcome as including the middle worms (concentration coefficient *p* < 0.05, neuron coefficient *p* < 0.05, data not shown). In AWA-mediated chemotaxis assays, worms migrated to both the middle and control regions (comparable positive parameter estimates and p-values) as MC-LR concentration increased, supporting the need to compare the odor endpoint to the combined middle and control endpoints as the two outputs for the model.

Toxin type and neuron type were used as additional explanatory variables and to identify interaction terms. Parameter estimates are presented in log odds ratio. Data in boxplots, (bold horizontal bar in the middle of the box is the median value, the bottom and top of the box represent the 25th and 75th percentiles, respectively, and whiskers extend to the farthest data point within 1.5 interquartile ranges from the edges of the box, with extreme values separated as circles) are presented as the proportion of worms at the odor (number of worms at odor/(number of worms at odor + number of worms at control + number of worms at middle)) on the y-axis, and toxin concentration on the x-axis. Significance was attributed to *p* < 0.05 *, *p* < 0.01 ** and *p* < 0.001 ***.

## 4. Conclusions

Using the statistical method established in this paper, the chemotaxis assay is a sensitive approach for detecting neuron-specific toxicity over a range of toxin concentrations. Thus, this assay demonstrated that MC-LR and MC–LF selectivity targeted adult *C. elegans* AWA sensory neurons in the absence of severe systemic toxicity compared to AWC sensory neurons. It is possible that MCs target other neurons required for AWA function, but not AWC function, resulting in altered AWA-mediated behavior. Although the AWC has been demonstrated to play a minor role in chemotaxis at high diacetyl concentrations [37], our data suggests that at low diacetyl concentrations the AWC does not play a role in chemotaxis to diacetyl. Another hypothetical assumption to consider is that the functional AWC neuron masked some of the AWA impairment via cellular redundancy between olfactory neurons. Initial experiments using tautomycin and okadaic acid suggest that MCs do not alter AWA function through inhibition of PP1 or 2A. The observation that MC neurotoxicity exhibits an inverted concentration-relationship effect on AWA-mediated chemotaxis has important implications for MC risk assessments. While the ultimate goal is to reduce and prevent MC exposure, their immediate threat on human and animal health needs to be fully understood. *C. elegans* is a useful model for predicting neurotoxic effects in mammals [69], and with this study, this cost effective and simple model is better established as a suitable platform to further investigate the mechanism(s) of MC’s neurotoxicity and screen the relative neurotoxic potency of the different MC isoforms.

The concentrations of MC-LR and MC-LF we used were based on previously published work [40,41] and are environmentally relevant. Total MCs exceeding 2000 µg/L have been detected in surface water bodies, and these levels are associated with toxicity [70]. The concentrations of MCs, tautomycin and okadaic acid taken up by *C. elegans* neurons in this study are unknown, as are the metabolism, distribution and excretion rates of these PP inhibitors once taken up by the worm. Therefore, we cannot determine whether MCs, tautomycin and okadaic acid have different impacts on worm behavior due to bioavailability, different PP inhibitory constants, or through mechanisms of action unrelated to PP inhibition.

Our exposure model assumes uptake of toxins via ingestion and subsequent distribution into neurons by OATPs, as the worm cuticle is very resilient [71] and the AWA and AWC sensory neuron endings are buried in the sheath and not exposed through the amphid pore [33]. Currently, there are no data on the presence and/or distribution of OATPs in *C. elegans* sheath cells and neurons. OATPs could serve as a mechanism for uptake of MCs through the pore and into the olfactory neurons. In mammals, MC exposure occurs primarily through ingestion, though other routes, such as inhalation [72], can occur. OATPs, specifically OATP1B1 and OATP1B3, play a major role in MC uptake into target cells, as they do not cross cell membranes easily [16,73]. Most *in vivo* MC exposures in mammalian models are done via intraperitoneal or intracerebroventricular injections; therefore, our oral and continuous exposure method may better reflect environmental exposure. Studies to investigate the role of calcium disruption in AWA impairment will help assess the risk of sub-acute MC exposures to contribute to neurological alterations and disease.
